# Functional Brain Network Abnormalities and Aripiprazole's Modulatory Effects in Pediatric Tic Disorder

**DOI:** 10.1002/brb3.71331

**Published:** 2026-03-30

**Authors:** Yuxin Xiang, Yilei Yao, Lin Jiang, Dan Sun, Jun Jiang, Zhisheng Liu

**Affiliations:** ^1^ Department of Neurology, Wuhan Children's Hospital, Tongji Medical College Huazhong University of Science and Technology Wuhan China; ^2^ University of Electronic Science and Technology of China Chengdu China; ^3^ Department of Electrophysiology Laboratory, Wuhan Children's Hospital, Tongji Medical College Huazhong University of Science and Technology Wuhan China

**Keywords:** aripiprazole, functional brain network, functional connectivity, network topological properties, pediatric tic disorder

## Abstract

**Background:**

This study aimed to investigate functional brain network characteristics in pediatric tic disorder (TD) patients and explore the effects of aripiprazole treatment on the functional brain network.

**Methods:**

Resting‐state electroencephalogram (EEG) was recorded from 40 TD patients and 30 matched healthy controls to compare functional brain network differences between TD patients and healthy individuals. Additionally, 26 of these TD patients underwent a 12‐week aripiprazole treatment and were evaluated for changes in their functional brain networks before and after treatment. Functional connectivity between brain regions was analyzed using the phase locking value (PLV), and global network topological properties (global efficiency, local efficiency, clustering coefficient, and characteristic path length) were quantified via graph theory.

**Results:**

Compared with controls, TD patients exhibited reduced frontal functional connectivity (Fp2–Fz, Fp2–F4, Fz–F4) but compensatory enhanced connectivity in F7–F3 and C3–Cz. Tourette syndrome patients further exhibited Fp1–Fz hypo‐connectivity. Aripiprazole treatment significantly increased global functional connectivity, elevated global and local efficiency and clustering coefficient, and shortened characteristic path length in TD patients. However, frontal hypo‐connectivity persisted after treatment partly.

**Conclusions:**

Pediatric TD patients present frontal dysfunction with compensatory adaptations in frontal–temporal connection and sensorimotor networks. Aripiprazole modulates global brain network connectivity and topology but not does not normalize frontal deficits, providing neurophysiological evidence for TD pathophysiology and aripiprazole's therapeutic mechanism.

## Graphical abstract

Pediatric tic disorder patients show frontal functional hypo‐connectivity with compensatory network adaptations. Aripiprazole modulates global brain network connectivity and topology but fails to normalize frontal deficits, providing neurophysiological insights into TD pathophysiology and the drug's therapeutic mechanism.

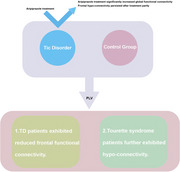

**Abbreviations**
ADHD: attention‐deficit/hyperactivity disorder
CBIT: comprehensive behavioral intervention for tics
CSTC: cortico‐striato‐thalamo‐cortical
CTD: chronic motor or vocal tic disorders
*DSM‐5*
: *Diagnostic and Statistical Manual of Mental Disorders, Fifth Edition*
EEG: electroencephalogram
FDR: false discovery rate
FPC: frontopolar cortex
FPN: frontoparietal network
ICA: independent component analysis
OCD: obsessive‐compulsive disorder
PFC: prefrontal cortex
PLV: phase locking value
PMC: premotor cortex
PTD: provisional tic disorders
REST: reference electrode standard technique
SMA: supplementary motor area
SMN: sensorimotor network
TD: tic disorder
TS: Tourette syndrome
YGTSS: Yale Global Tic Severity Scale

## 1 Introduction

Tic disorders (TD) are one of the common neurodevelopmental disorders, categorized into provisional tic disorders (PTD), chronic motor or vocal tic disorders (CTD), and Tourette syndrome (TS). TDs are characterized by sudden, rapid, recurrent, nonrhythmic movements or vocalizations, including eye blinking, facial grimacing, throat clearing, and even coprolalia or echolalia (Liu et al. 2020). They frequently co‐occur with comorbidities such as attention‐deficit/hyperactivity disorder (ADHD), obsessive‐compulsive disorder (OCD), anxiety disorder, and depressive disorder. The typical onset of tics occurs between 4–7 years, reach their peak at 10–12 years, and tend to diminish after puberty (Liu et al. 2020; Mataix‐Cols et al. 2023).

The pathogenesis of TDs remains incompletely understood. Current evidence indicates that the pathophysiology may involve functional abnormalities in the cortico‐striato‐thalamo‐cortical (CSTC) circuit (Ramteke and Lamture 2022). Additionally, a multifactorial interplay of genetic, neurophysiological, immunological, and environmental factors may contribute to the development of TD (Liu et al. 2020). With advances in human connectome research, many neurodevelopmental disorders are associated with aberrant structural and functional brain network connectivity (van den Heuvel and Sporns 2019). Studies have shown that TD brain networks exhibit altered network properties and disruptions in functional and structural connectivity, with these abnormalities closely correlated to symptom severity (Yang et al. 2023; Openneer et al. 2020). Using resting‐state functional MRI, Church et al. (2009) found that early‐to‐mid adolescent TS patients displayed immature functional connectivity, primarily within the frontoparietal network (FPN), when compared with age‐matched controls (Church et al. 2009). Similarly, Shprecher et al. (2014) reported increased connectivity between ipsilateral anterior/mid‐cingulate cortex and striatum, enhanced local connectivity, and reduced long‐range connectivity in males with TS (Shprecher et al. 2014). Collectively, these findings reflect immature functional connectivity patterns in TD patients.

Electroencephalogram (EEG) is a noninvasive, portable technology with high temporal resolution, making it a powerful tool for investigating electrical activity underlying brain function (Singh and Krishnan 2022). When combined with standardized analysis techniques, EEG can not only illuminate physiological processes but also pathophysiological alterations associated with neurological disorders (Yang et al. 2023). For example, Lee et al. (2021) reported that children with TD exhibited reduced posterior alpha activity compared to those with ADHD, using eLORETA analysis of resting‐state scalp‐recorded electrical potentials (Lee et al. 2021). Given that posterior alpha activity is associated with cortico‐thalamic network function, this finding in TD patients is consistent with dysfunction in the CSTC circuit (Ibarra‐Lecue et al. 2022).

While studies have identified abnormalities in functional brain networks in TD, these findings remain poorly translated into clinical practice. For instance, one study on comprehensive behavioral intervention for tics (CBIT) demonstrated that baseline functional connectivity related to tic suppression specifically predicted the reduction in vocal tic severity following treatment (Morand‐Beaulieu S, Crowley, et al. 2023). For pharmacotherapy, aripiprazole, a dopamine D2 receptor partial agonist, is a second‐generation antipsychotic recommended as first‐line pharmacotherapy for TDs. Feng et al. (2024) found that 12‐week aripiprazole treatment significantly attenuated progressive functional connectivity decline in subcortical, default mode, and other networks in schizophrenia patients (Feng et al. 2024). Nevertheless, despite established functional dysconnectivity in TDs, evidence regarding the effects of aripiprazole on functional connectivity in TDs remains limited.

In the study, to explore the change of brain function network in pediatric TD patients, we use EEG signals to construct functional brain networks in pediatric TD patients. Graph theory, a tool for analyzing the topological properties of complex networks, enables the quantification of network characteristics, and these metrics provide insights into brain network abnormalities (Openneer et al. 2020; Hallett et al. 2020). Given aripiprazole's efficacy in improving clinical symptoms in TD patients, we hypothesize that aripiprazole treatment will modulate functional brain networks and alter their topological properties.

## 2 Materials and Methods

### 2.1 Participants

Eligible participants were identified via the *Diagnostic and Statistical Manual of Mental Disorders, Fifth Edition* (*DSM‐5*). Additional inclusion criteria were as follows: (1) aged 4–18 years, regardless of gender; (2) no prior pharmacological or behavioral intervention at the time of enrollment; (3) normal results in imaging examinations; and (4) informed consent obtained from both parents and the child. Exclusion criteria included (1) children with global developmental delay/intellectual disability or psychiatric disorders; (2) those who had already received pharmacological or behavioral interventions at enrollment; (3) abnormal findings in imaging examinations; and (4) individuals with hepatic or renal dysfunction. Written consent was obtained from participants and their parents. Finally, 40 children with TD were recruited from Wuhan Children's Hospital.

The healthy control groups were selected from healthy children at the Pediatric Health Care Clinic. Inclusion criteria were as follows: (1) matched with the TD group in age and gender; (2) no neurological diseases, global developmental delay/intellectual developmental disorder or psychiatric disorders (assessed primarily through medical history taking [including family history inquiry], physical examination, and communications with children); (3) no history of taking any type of neuropsychiatric drugs; (4) normal findings in EEG and cranial imaging examinations; and (5) informed consent obtained from both parents and the child. Finally, 30 healthy children were included as the control group.

The protocol for the study was carefully approved by the Ethics Committee of Wuhan Children's Hospital (No. 2025R013‐E01) according to *Measures for Ethical Review of Life Sciences and Medical Research Involving Humans* in China.

To evaluate the severity of TD, we employed the Yale Global Tic Severity Scale (YGTSS), a clinician‐rated instrument widely used to evaluate tic severity and related functional impairment. It assessed motor and phonic tics across five domains: number, frequency, intensity, complexity, and interference, in addition to overall impairment in daily functioning (Wen et al. 2021).

Of the 40 children with TD, 26 children were enrolled in a 12‐week trial of aripiprazole. Aripiprazole was administered using a flexible dosing regimen, starting at 1.25–2.50 mg per day and titrated in 1.25–2.5 mg increments (Roessner et al. 2022). The final dose was individualized according to therapeutic response and adverse effects, with a maximum allowed dose of 30 mg/day. During the 12‐week trial, no antitic medications other than aripiprazole were permitted.

Throughout the 12‐week treatment period, telephone follow‐ups were conducted for the pediatric patients every 2 weeks to monitor adverse drug reactions and reinforce medication adherence, and outpatient follow‐ups were scheduled every 4 weeks. At the 8th week, liver and renal function tests were carried out for the patients to screen for adverse reactions related to medication. All 26 pediatric patients demonstrated good treatment adherence (defined as no more than one missed dose of the prescribed medication within a 10‐day period) without dropouts throughout the study.

### 2.2 EEG Acquisition

EEG data were collected using the Nihon Kohden EEG‐1200C system. Electrodes were placed at positions (Fp1, Fp2, F3, F4, F7, F8, FZ, C3, C4, CZ, P3, P4, PZ, P7, P8, O1, O2, T7, T8) according to the international 10–20 system standards. Participants underwent a 10–15 min resting‐state recording with eyes open, during which they remained awake and were not instructed to suppress tics. EEG data were sampled at 500 Hz^5^. Electrode impedance was always kept below 5 kΩ.

### 2.3 EEG Preprocessing

All preprocessing steps were conducted in MATLAB. First, a 1–45 Hz bandpass filter was applied. Then, EEG signals were segmented into epochs with a duration of 5 s, followed by baseline correction. Bad channels were then identified via visual inspection and interpolated using spherical spline interpolation. Next, independent component analysis (ICA) was performed to isolate and remove artifacts related to eye blinks, muscle activity, and line noise, leveraging a combination of automated clustering algorithms and manual component inspection (Castells et al. 2007). Manual visual rejection was used to eliminate residual uncorrectable artifacts. Finally, the final retention rate of the EEG data was 76.5% (criterion for exclusion: 5‐s epochs with an artifact ratio exceeding 20% were excluded). Additionally, the reference electrode standard technique (REST) was employed to eliminate potential biases caused by the selection of reference electrodes (Chaddad et al. 2023). Subsequently, the EEG signals were decomposed into five canonical frequency bands—delta (1–4 Hz), theta (4–8 Hz), alpha (8–13 Hz), beta (13–30 Hz), and gamma (30–45 Hz)—using Welch's method.

### 2.4 Functional Connectivity Analysis

The method of connectivity analysis was based on phase locking value (PLV). PLV is a nonlinear analytical method based on phase synchronization. It can characterize the dynamic properties of coordinated activity in brain networks by quantifying the statistical consistency of instantaneous phases between two signals, and has been used to assess neural signal synchrony at electrode sites (Dirik 2025).

### 2.5 Network Measures

Based on PLV, we constructed brain functional connectivity networks and characterized their topological properties using four graph theory metrics. Global efficiency is defined as the mean of global efficiency across all regions and reflects the global efficiency of parallel information transfer in a network (Yang et al. 2023; Wen et al. 2018). Local efficiency measures the communication efficiency among a given node's first neighbors (after the node is removed) and reflects local information processing capability by quantifying the transfer efficiency of shortest paths among the node's neighbors (Vecchio et al. 2017; Rubinov and Sporns 2010). Clustering coefficient is the average over all nodes, reflecting the degree of local interconnectivity and clustering in a network (Wen et al. 2018). Characteristic path length measures the average distance or routing efficiency between any pair of network nodes, with higher values indicating lower routing efficiency (Liao et al. 2022).

## 3 Statistical Analysis

Statistical analysis and processing were performed using the MATLAB toolbox. Continuous data were expressed as mean ± standard deviation (mean ± SD) or median (25th percentage, 75th percentage), and categorical data were expressed as frequency (percentage). Group comparisons were conducted using the *t*‐test for normally distributed continuous data and the Wilcoxon test (Mann–Whitney *U* test or Wilcoxon signed‐rank test) for non‐normally distributed continuous data. The chi‐square test was used for categorical data. All statistical tests were corrected for multiple comparisons across the full frequency band (1–45 Hz) and each of the five canonical frequency bands using the false discovery rate (FDR) method. A *p* value < 0.05 was considered statistically significant.

## 4 Results

### 4.1 Demographic Features

A total of 40 children with TD (36 males and 4 females) and 30 controls (27 males and 3 females) were included in this study. They were matched for gender (males/females: 36/4 patients and 27/3 controls; *p* > 0.05) and age (years, mean ± SD: 8.83 ± 2.27 patients and 8.90 ± 3.09 controls, *p* = 0.87) (Table 1). The detailed demographic of the TD subgroups are shown in Table 2.

**TABLE 1:** Baseline characteristics of children with tic disorders and the control group.

Baseline characteristics	Tic disorder patients	Controls	*p* value
Number of patients	40	30	
Males:females^a^	36:4	27:3	> 0.05
Age (mean ± SD)^b^	8.83 ± 2.27	8.90 ± 3.09	0.87
Syndrome duration (month, median [25th, 75th])	24 (12, 36)		
Global score of Yale Global Tic Severity Scale (score, mean ± SD)	24.90 ± 7.04		
Motor score	10.67 ± 3.95		
Vocal score	5.90 ± 5.49		
Impairment score	9.00 ± 3.03		

^a^ Chi‐square test.

^b^ Mann–Whitney *U* test.

**TABLE 2:** Detailed demographics of children with tic disorders subgroups.

TD subgroups	Demographics	
Provisional tic disorders	Number of patients	9
	Males:females	9:0
	Age (year, mean ± SD)	8.19
	Global score of Yale Global Tic Severity Scale (score, mean ± SD)	23.44 ± 2.22
Chronic tic disorders	Number of patients	7
	Males:females	4:3
	Age (year, mean ± SD)	8.29 ± 2.09
	Global score of Yale Global Tic Severity Scale (score, mean ± SD)	19.71 ± 5.75
Tourette syndrome	Number of patients	24
	Males:females	23:1
	Age (year, mean ± SD)	9.24 ± 2.39
	Global score of Yale Global Tic Severity Scale (score, mean ± SD)	27.92 ± 6.38

A 12‐week course of aripiprazole was administered to 26 children (mean daily dose: 6.38 ± 2.13 mg). The YGTSS score was reassessed after treatment (before vs. after): motor score (10.67 ± 3.95 vs. 9.23 ± 2.87, *p* = 0.20), vocal score (5.90 ± 5.49 vs. 4.10 ± 4.61, *p* = 0.01), impairment score (9.00 ± 3.03 vs. 8.92 ± 6.28, *p* = 0.76), Global YGTSS score (24.90 ± 7.04 vs. 22.35 ± 9.84, *p* = 0.18).

### 4.2 Functional Connectivity

**FIGURE 1:**
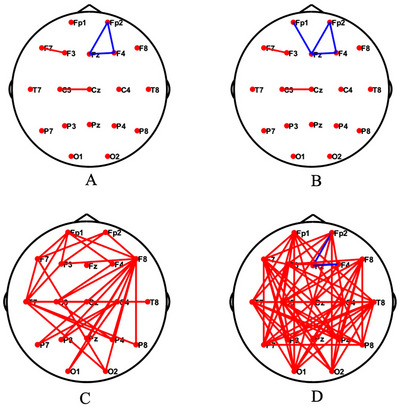
Functional connectivity analysis based on phase locking value. (A) TD group versus control group, (B) TS group versus control group, (C) post‐aripiprazole treatment group versus pre‐aripiprazole treatment group, and (D) post‐aripiprazole treatment group versus control group. *Red connections indicate stronger functional connectivity in the former group compared to the latter, while blue connections indicate weaker functional connectivity in the former group compared to the latter.

Compared with the control group, TD patients exhibited significantly weaker functional connectivity measured by PLV in several frontal connection: between Fp2 and Fz (*t* = −2.52, *p* = 0.01), Fp2 and F4 (*t* = −2.24, *p* = 0.01), and Fz and F4 (*t* = −2.14, *p* = 0.02) (Figure 1). Additionally, a notably reduced connectivity was observed between Fp1 and Fz in TS patients (*t* = −4.04, *p* < 0.01). In contrast, both TD and TS patients displayed increased functional connectivity between F7 and F3 (TDs vs. controls: *t* = 2.19, *p* = 0.02; TS vs. controls: *t* = 2.50, *p* = 0.01) as well as between C3 and Cz (TDs vs. controls: *t* = 2.46, *p* = 0.01; TS vs. controls: *t* = 2.31, *p* = 0.01). Differences in functional connectivity across other frequency bands are presented in Supporting Information S1. The results of power spectral density analysis are also presented in Supporting Information S1.

Compared with the pre‐aripiprazole treatment group, the post‐aripiprazole treatment group showed significantly increased brain functional connectivity, with no functional connectivity being decreased. Furthermore, compared with the control group, the aripiprazole treatment group exhibited a tendency toward increased brain functional connectivity, while the decreased functional connectivity remained (from Fp2 to Fz [post‐aripiprazole treatment group vs. controls: *t* = −2.07, *p* = 0.02] and from F4 to Fz [post‐aripiprazole treatment group vs. controls: *t* = −2.70, *p <* 0.01]).

### 4.3 Topological Properties

**FIGURE 2:**
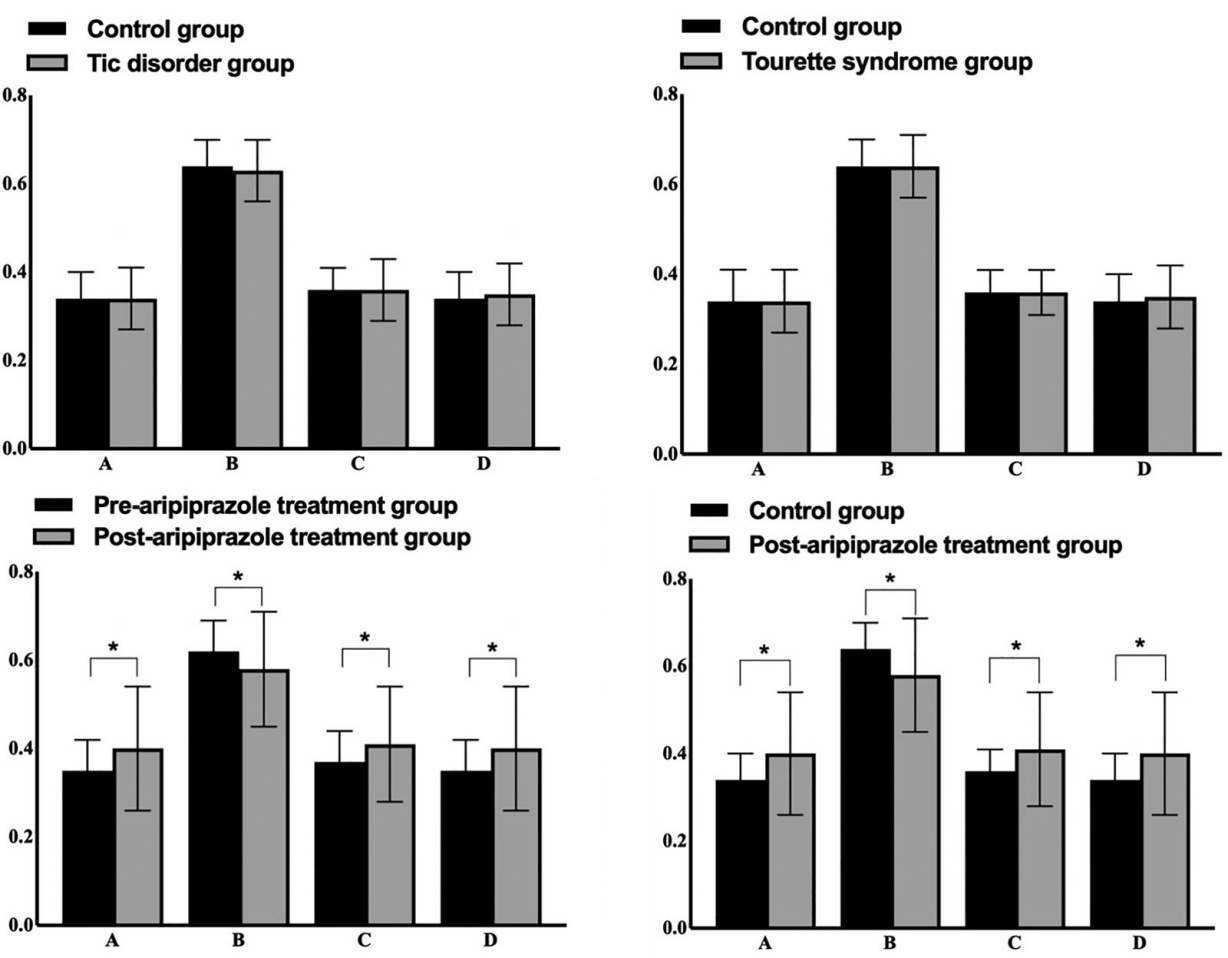
Analysis results of topological properties. (A) Clustering coefficient, (B) characteristic path length, (C) global efficiency, and (D) local efficiency. ^*^ *p* < 0.05.

Global topological properties showed no significant difference between the TD and control groups. Similarly, no significant difference in global topological properties was observed between the TS and control groups. However, compared with TD patients before treatment, those after treatment exhibited slightly higher topological properties: global efficiency (0.37 ± 0.07 vs. 0.41 ± 0.13, *p* = 0.03), local efficiency (0.35 ± 0.07 vs. 0.40 ± 0.14, *p* = 0.03), and clustering coefficient (0.35 ± 0.07 vs. 0.40 ± 0.14, *p* = 0.03). In contrast, the characteristic path length was shorter in the posttreatment group (0.63 ± 0.07 vs. 0.58 ± 0.13, *p* = 0.03). Additionally, these posttreatment topological property values in TD patients also showed significant differences from those of the control group, specifically characterized by higher global efficiency (0.41 ± 0.13 vs. 0.37 ± 0.07, *p* = 0.01), higher local efficiency (0.40 ± 0.14 vs. 0.35 ± 0.07, *p <* 0.01), higher clustering coefficient (0.40 ± 0.14 vs. 0.34 ± 0.06, *p <* 0.01), and a shorter characteristic path length (0.37 ± 0.07 vs. 0.41 ± 0.13, *p* = 0.03) (Figure 2).

## 5 Discussion

Functional network abnormalities have been widely reported in children with TD. In the present study, we observe reduced functional connectivity between Fp2–Fz, Fp2–F4, and Fz–F4 primarily within frontal lobe. The frontal lobe, a key region for executive control, contains critical nodes of the inhibitory neural network (Zea Vera et al. 2022; Xie et al. 2026). Previous research has demostrated that enhancing frontal lobe activities may facilitate inhibitory control over impulsive behaviors (Krishna et al. 2025). In typically developing children, the frontal lobe exerts effective inhibitory control over hyperexcitability in the primary motor cortex (M1), whereas studies have demonstrated that this inhibitory function is impaired in children with CTD, and the more severe the inhibitory deficits, the more difficult it is to suppress tics (Bruce et al. 2021). Consistent with our findings, Cheng et al. (2013) used diffusion tensor imaging to demonstrate that the connectivity of cortico‐cortical circuits in the frontal lobes of patients with TS is reduced, confirming the existence of abnormal structural connectivity of frontal lobe in TS patients (Cheng et al. 2013). Similarly, our study identified reduced connectivity in Fp2–Fz, Fp2–F4, and Fz–F4 in TD patients, along with a further weakening of the Fp1–Fz pathway in TS patients. These findings confirm that frontal lobe connectivity aberrations represent a critical neural feature of TD, and the further reduction in frontal pathway connectivity specific to TS patients may underpin the more severe manifestations and more pronounced inhibitory control deficits in this subgroup.

Moreover, as the brain's executive hub for higher‐order cognition, the prefrontal cortex (PFC) exhibits neuroplasticity that is directly associated with the regulation of complex behaviors (Nie and Fan 2025). As the supreme integrative hub of PFC, the frontopolar cortex (FPC) serves as a critical region of the PFC, responsible for multimodal information integration, complex decision‐making, and regulation of goal‐directed behaviors (Nie and Fan 2025; Chen et al. 2023). Notably, studies have indicated that the thinner cortex of the dorsomedial PFC is associated with more severe tic symptoms (Sowell et al. 2008). Given that the FPC plays a key role in integrating multimodal information to support cognitive control including inhibitory regulation and adaptive attention shifts, reduced connectivity between the FPC and other frontal regions may further explain tic suppression difficulties in TD children.

Interestingly, we also identify strengthened connectivity between F7 and F3. This finding is similar to a previous study reporting greater involvement of the left dorsal medial PFC in functional networks of TD patients, which is interpreted as a potential compensatory response to reduced frontal connectivity (Fan et al. 2018). Previous studies have often shown that in neurological and psychiatric diseases, brain networks undergo adaptive and compensatory changes (Suo et al. 2026; Suo et al. 2025). Therefore, in the present study, the enhanced connectivity in the left frontal lobe may represent a compensatory mechanism developed by TD patients as a result of long‐term tic suppression. However, due to the inherent limitation of this study's cross‐sectional design, we cannot determine whether the compensatory network change is the consequence of long‐term tic suppression or a preexisting neural trait. Future longitudinal studies tracking brain network dynamics and tic symptoms over time, or interventional studies targeting this frontal connectivity, are needed to clarify the directional relationship.

Moreover, the presence of increased functional coupling is found from C3 to Cz. This is consistent with the studies which reported that inter‐network functional connectivity is increased in the sensorimotor network (SMN) (Tikoo et al. 2020; Xin et al. 2023). SMN includes the primary motor cortex (M1), the somatosensory cortex, the premotor cortex (PMC), and the supplementary motor area (SMA), which plays a crucial role in daily functioning, encompassing the integration of sensory and motor formation to execute purposeful movements (Sahrizan et al. 2025). Given that the onset of both motor and vocal tics has been linked to activation in sensorimotor regions (Hong et al. 2013), the heightened connectivity observed here may underlie neural activity associated with tic generation.

Furthermore, neuroimaging studies have reported that aripiprazole treatment reduces activation in several brain regions, including the middle and superior frontal gyri, occipital gyrus, medial temporal and inferior frontal gyri, putamen, and cuneus in patients with schizophrenia (Liemburg et al. 2017). Additionally, Feng et al. (2024) found that aripiprazole significantly decreased within‐ and between‐network functional connectivity in large‐scale networks in schizophrenia patients (Feng et al. 2024). These findings suggest that aripiprazole can modulate brain network activity. In the present study, aripiprazole treatment primarily enhances functional connectivity between brain regions, although it doesn't fully reverse the preexisting reductions in frontal connectivity. This is demonstrated by the posttreatment group still partly exhibiting lower frontal connectivity relative to the control group, while showing significantly increased global functional connectivity compared to the pretreatment baseline. Aripiprazole, a partial D_2_ agonism, is belived to stabilize dopamine and serotonin activity within the nucleus accumbens, ventral tegmental area, and frontal cortex (Tuplin and Holahan 2017). Because of high D_2_ affinity, aripiprazole maintains synaptic dopamine function at a constant submaximal level of activation regardless of local dopamine concentration. This results in functional antagonism in brain regions with high dopamine concentrations such as the striatum, whereas in areas with low dopamine concentrations like the frontal cortex, aripiprazole's partial agonism outweighs dopamine's local action (Murphy et al. 2016). Therefore, rather than repairing a specific circuit, aripiprazole may exert its therapeutic effects by regulating dopamine as a hub‐like regulatory system across the whole brain and by enhancing overall brain network integration.

According to previous studies, despite the common small‐world topology, TS children showed decreased global and local efficiency alongside increased characteristic path length (Wen et al. 2017). In contrast, our study failed to detect significant differences in global network properties between TD/TS patients and controls, a result consistent with the findings of Yang et al. (2023). However, this may not mean that the brain network properties of children with TD have undergone no alterations compared with typical children. In fact, in Yang's study, even though the global network properties remained unchanged, the local properties of some brain regions still exhibited notable alterations (Yang et al. 2023). Moreover, many previous studies reporting significant global topological differences between TD/TS patients and controls were based on MRI, which possesses substantially higher spatial resolution than EEG signals (Liao et al. 2022). This may be a key factor for the absence of significant global topological differences in our study.

Following aripiprazole treatment, we observe increases in the clustering coefficient, global efficiency, and local efficiency, accompanied by a decrease in characteristic path length. These topological improvements are consistent with the increased functional connectivity observed after treatment, suggesting that aripiprazole may enhance global information transfer efficiency and network stability. Notably, prior research has shown that tic suppression is associated with shortened characteristic path length and elevated global efficiency, which improve the directness and efficiency of information processing (Morand‐Beaulieu et al. 2023). This raises the question of whether aripiprazole alleviates tics through a similar mechanism, possibly by optimizing brain network topology to support improved inhibitory control.

This study has limitations. The sample size of patients before and after aripiprazole treatment included in this study is relatively small, which may increase the risk of false‐positive findings. More notably, because EEG signals have low spatial resolution, they cannot accurately identify exact anatomical locations, which means the interpretation of results regarding the direction of information flow requires caution. Besides, studies have demonstrated that structural–functional connectivity coupling, which quantifies the association between structural connectivity and functional connectivity, can detect subtle brain alterations with greater sensitivity than a single modality, even at the early stage of a disease (Suo et al. 2026; Suo et al. 2025). Therefore, future research can incorporate a broader array of brain function assessment metrics, particularly those derived from MRI modalities, to more comprehensively elucidate the neural mechanisms underlying TD.

## 6 Conclusion

Pediatric TD patients present frontal dysfunction with compensatory adaptations in frontal–temporal connection and SMNs. Aripiprazole modulates global brain network connectivity and topology but not does not normalize frontal deficits, providing neurophysiological evidence for TD pathophysiology and aripiprazole's therapeutic mechanism.

## Author Contributions

Writing – review and editing: Zhisheng Liu, Jun Jiang, and Dan Sun. Writing – original draft and formal analysis: Yuxin Xiang and Yilei Yao. Methodology: Lin Jiang. All authors have read and approved the manuscript.

## Funding

This work was supported by the National Key Research and Development Plan (2021YFC0863700) and the Hubei Provincial Science and Technology Plan Project for Clinical Research Center of Neurodevelopmental Disorders in Children (2022DCC020).

## Ethics Statement

The study was approved by Ethics Committee of Wuhan Children's Hospital (No. 2025R013‐E01).

## Conflicts of Interest

The authors declare no conflicts of interest.

## Supporting information




**Supplemetary Materials**: brb371331‐sup‐0001‐SuppMat.pptx

## Data Availability

The datasets generated during and/or analyzed during the current study are available from the corresponding author on reasonable request.
