# Supplementary material for: Functional Brain Network Abnormalities and Aripiprazole's Modulatory Effects in Pediatric Tic Disorder
Source: Brain Behav. 2026 Mar 30;16(4):e71331. doi: 10.1002/brb3.71331 (PMC13112030; doi:10.1002/brb3.71331)
Supplement: Supplementary file 1 — Supplemetary Materials: brb371331‐sup‐0001‐SuppMat.pptx [file BRB3-16-e71331-s001.pptx]

## Slide 1
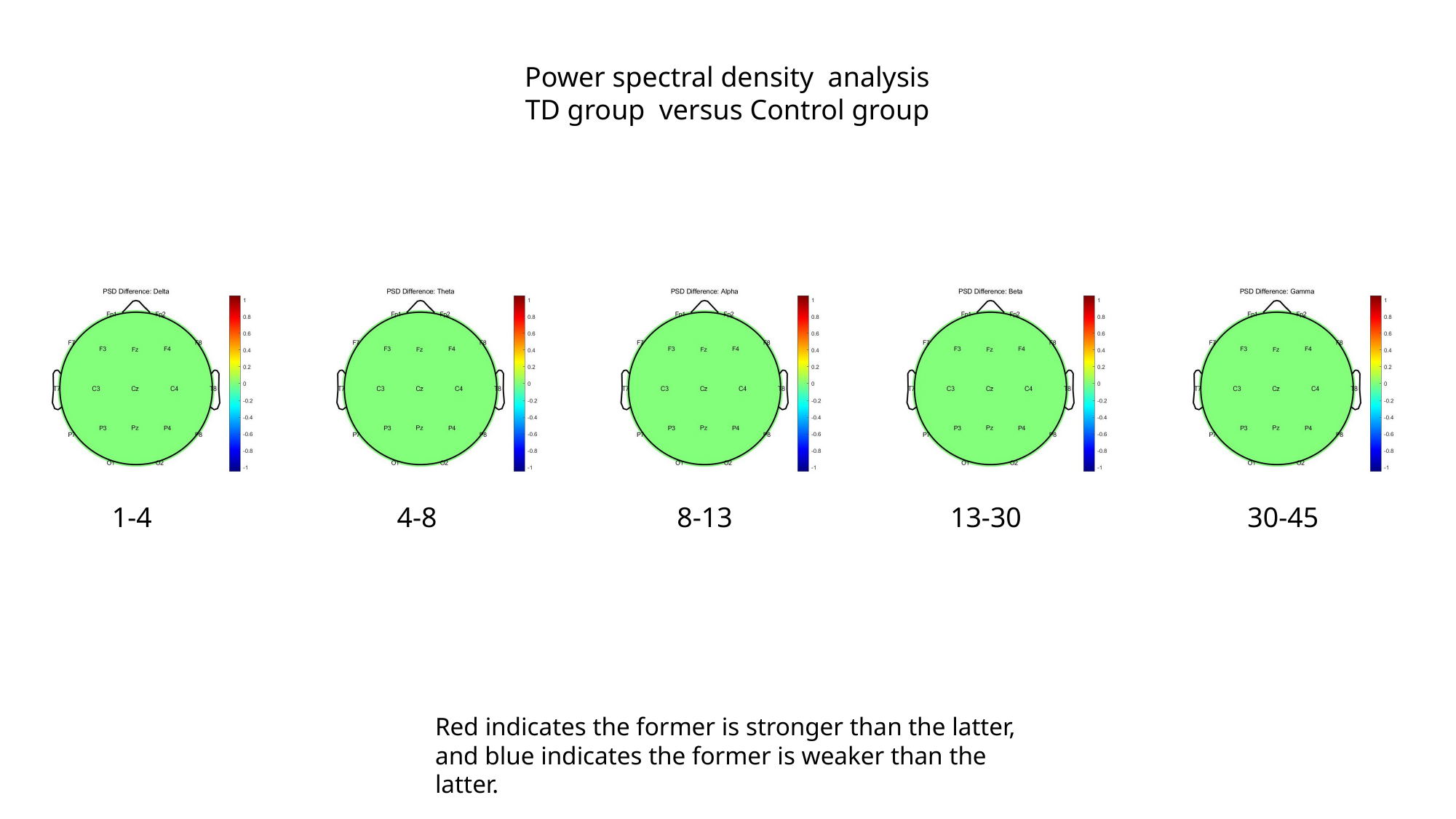

Power spectral density analysis
TD group versus Control group
1-4
4-8
8-13
13-30
30-45
Red indicates the former is stronger than the latter, and blue indicates the former is weaker than the latter.

## Slide 2
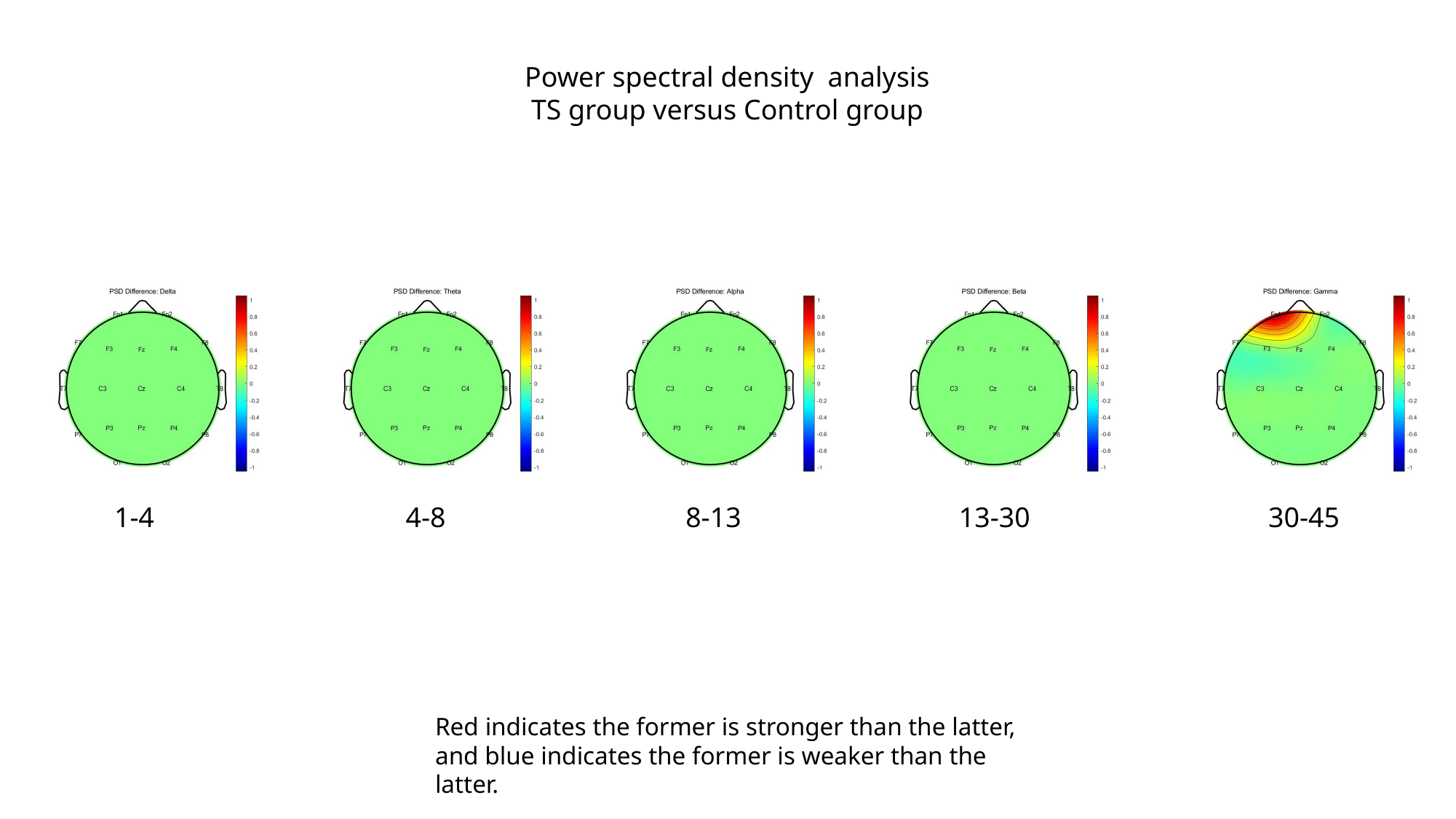

Power spectral density analysis
TS group versus Control group
1-4
4-8
8-13
13-30
30-45
Red indicates the former is stronger than the latter, and blue indicates the former is weaker than the latter.

## Slide 3
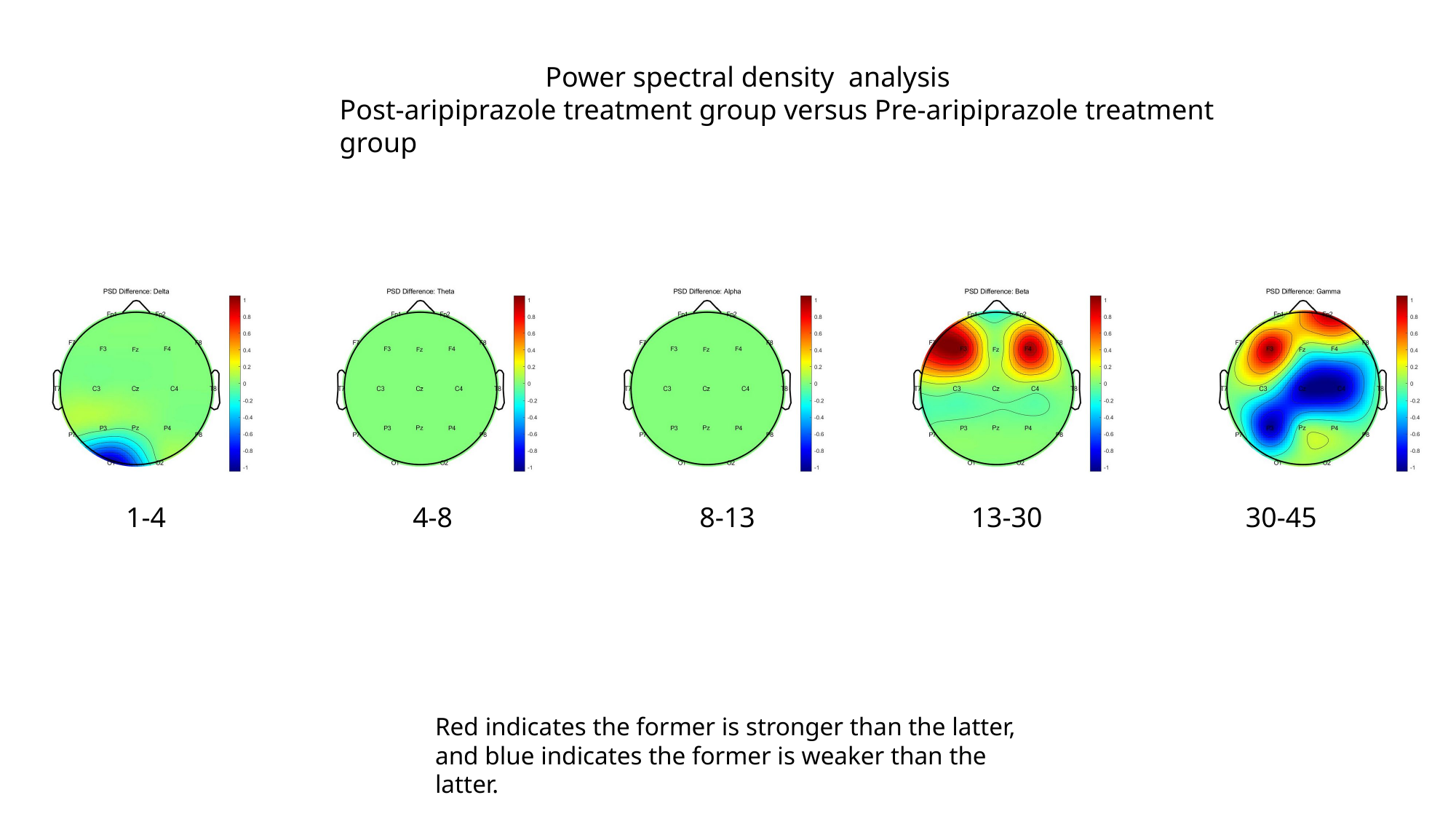

Power spectral density analysis
Post-aripiprazole treatment group versus Pre-aripiprazole treatment group
1-4
4-8
8-13
13-30
30-45
Red indicates the former is stronger than the latter, and blue indicates the former is weaker than the latter.

## Slide 4
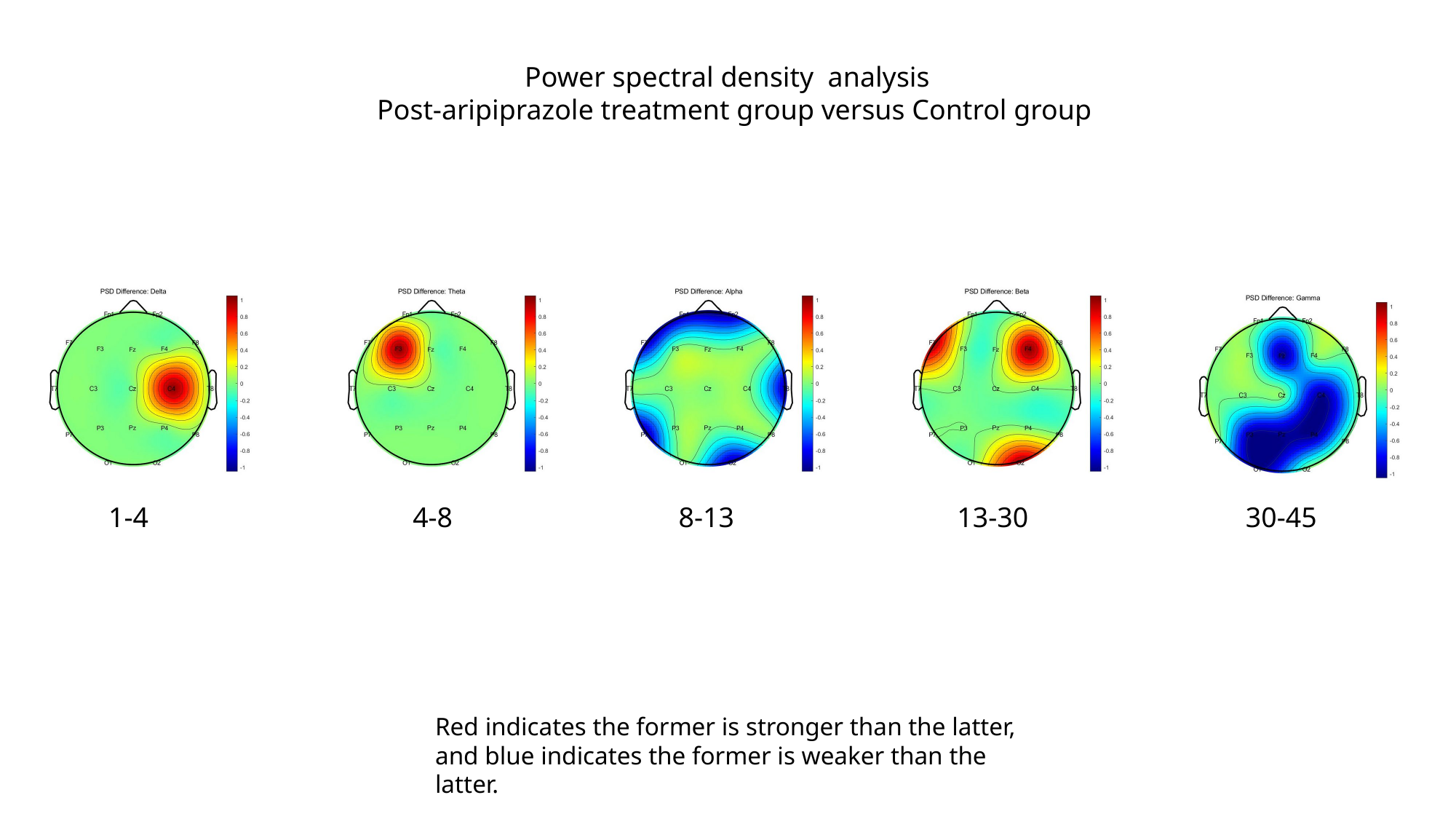

Power spectral density analysis
 Post-aripiprazole treatment group versus Control group
1-4
4-8
8-13
13-30
30-45
Red indicates the former is stronger than the latter, and blue indicates the former is weaker than the latter.

## Slide 5
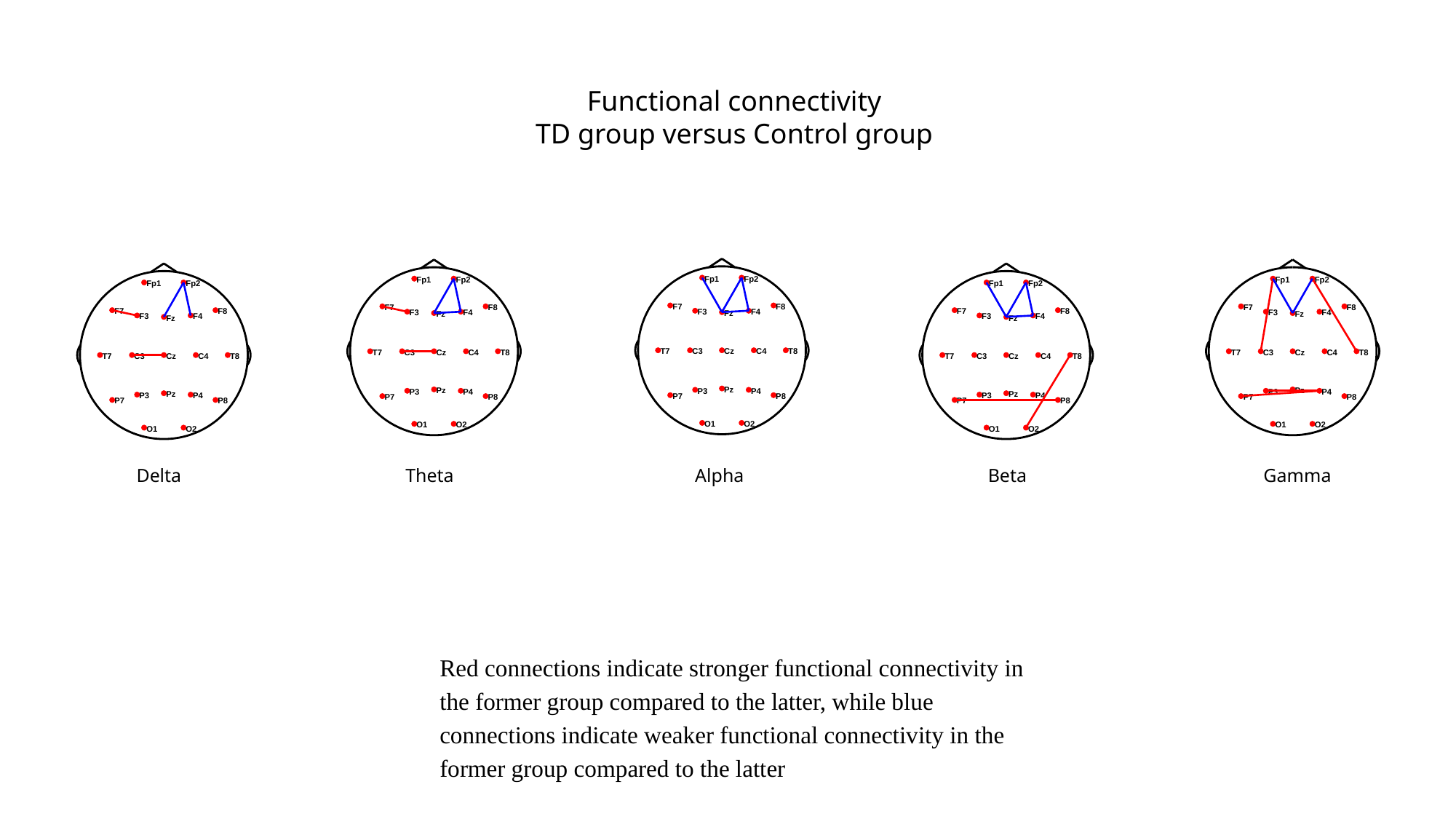

Functional connectivity
TD group versus Control group
Gamma
Theta
Alpha
Beta
Delta
Red connections indicate stronger functional connectivity in the former group compared to the latter, while blue connections indicate weaker functional connectivity in the former group compared to the latter

## Slide 6
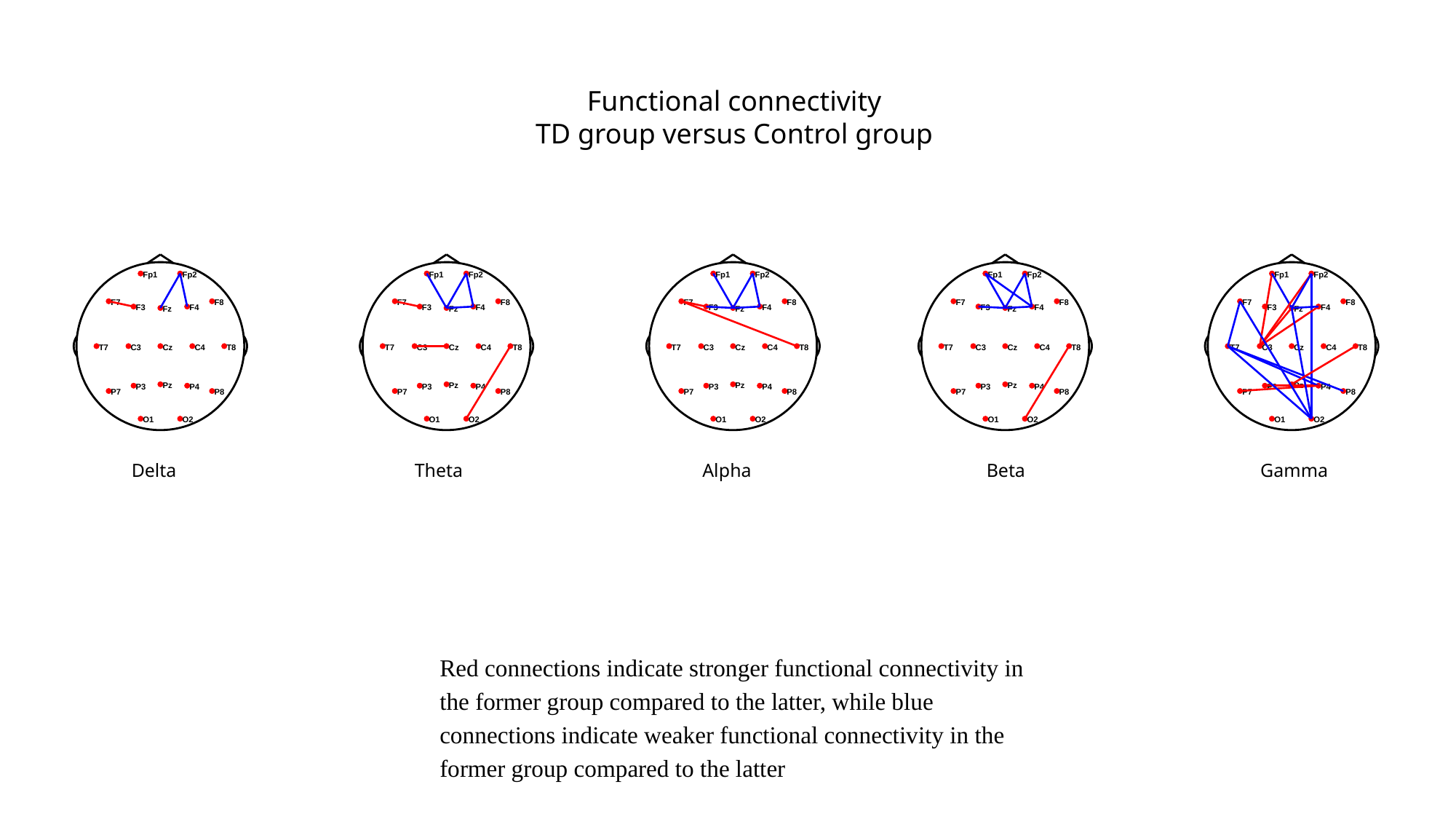

Functional connectivity
TD group versus Control group
Gamma
Theta
Alpha
Beta
Delta
Red connections indicate stronger functional connectivity in the former group compared to the latter, while blue connections indicate weaker functional connectivity in the former group compared to the latter

## Slide 7
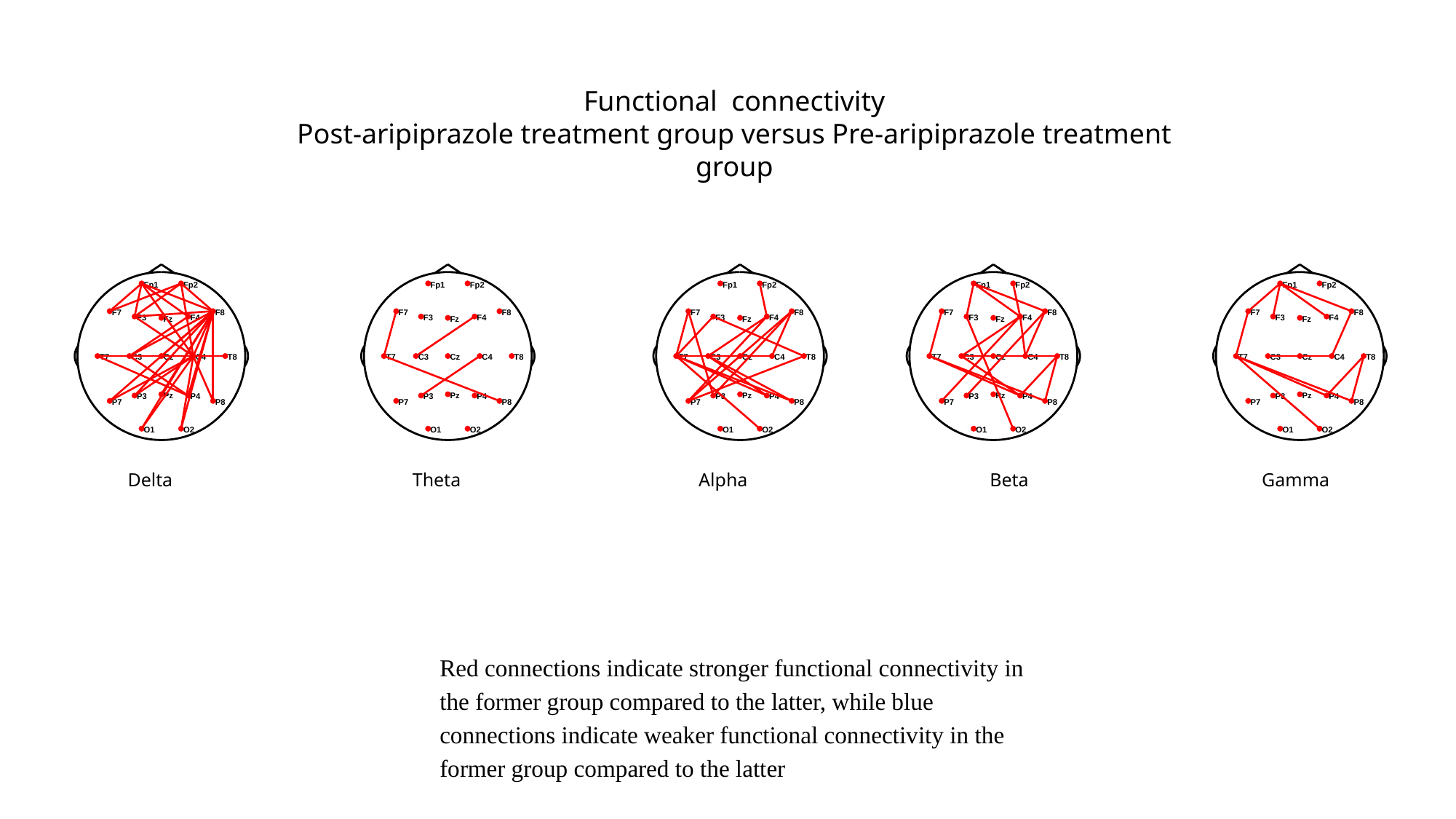

Functional connectivity
Post-aripiprazole treatment group versus Pre-aripiprazole treatment group
Gamma
Theta
Alpha
Beta
Delta
Red connections indicate stronger functional connectivity in the former group compared to the latter, while blue connections indicate weaker functional connectivity in the former group compared to the latter

## Slide 8
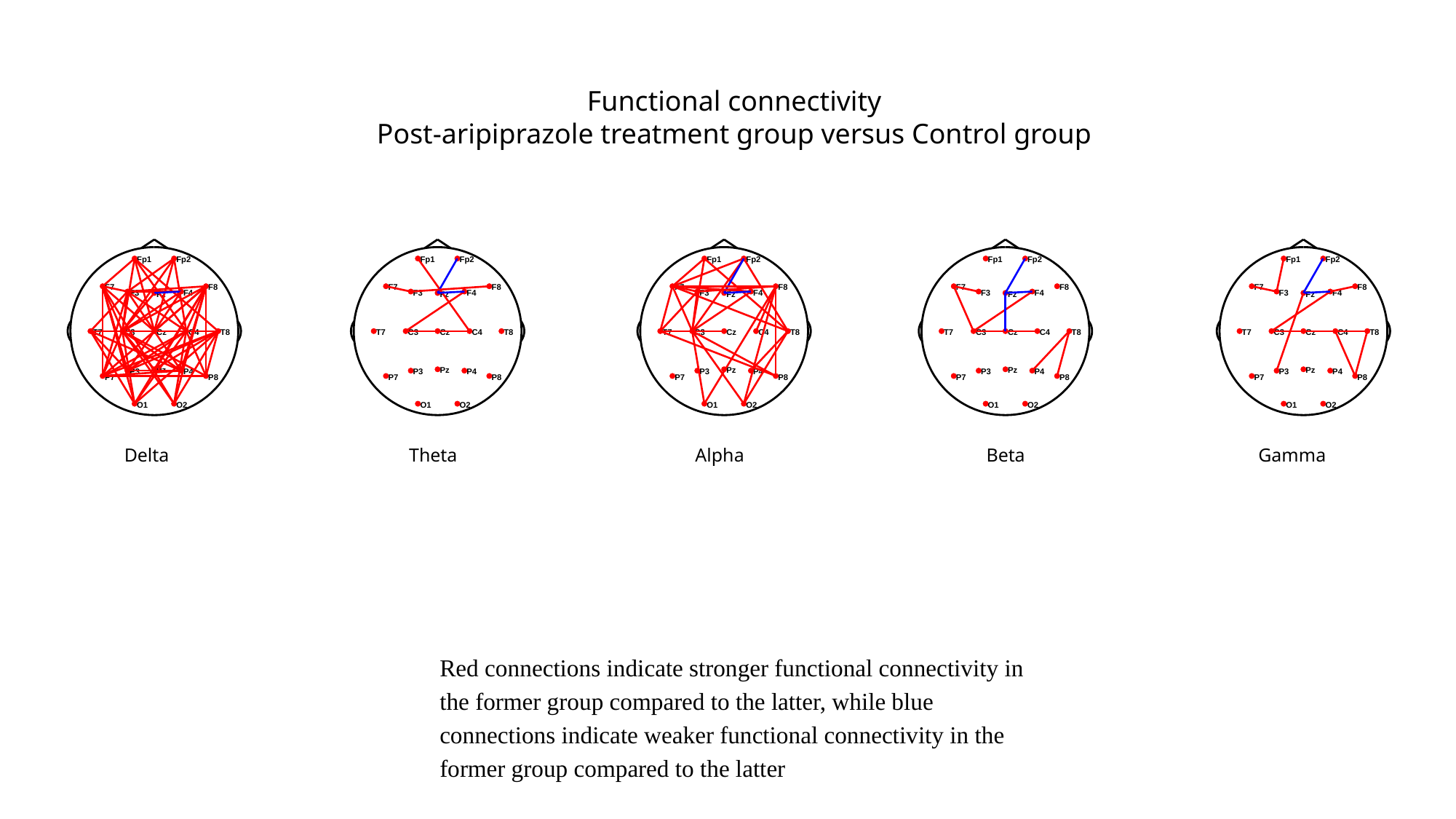

Functional connectivity
Post-aripiprazole treatment group versus Control group
Gamma
Theta
Alpha
Beta
Delta
Red connections indicate stronger functional connectivity in the former group compared to the latter, while blue connections indicate weaker functional connectivity in the former group compared to the latter
